# Book Reviews

**Published:** 1918-10

**Authors:** 


### BOOK REVIEWS

Books mentioned may be inspected at and ordered through this office. So far as possible, books received in any month will be reviewed in the issue of the second month following. Pamphlets, quarterly and similar periodicals, reports, transactions, etc., will, as a rule, merely be mentioned.

---

**On the Fringe of the Great Fight,** by Colonel George G. Nasmith, C. M. G. George H. Doran Co., Publishers, 244 Madison Ave., New York City. Price, $1.50.

This book tells of some of the experiences of the first Canadian Contingent. The narrative starts at the first training camp at Valcartier and further tells of the camp on Salisbury Plains in England and trench warfare in France. Physicians will find the detailed account of the mobile laboratory work especially fascinating, and will be surprised at the vast field of this department.

The author's fine sense of humor and splendid literary style, plus his scientific training, make this book one of the best war accounts published to date.

---

**The Medical Clinics of North America.** Volume 1. Number 6. (The Southern Number, May 1918). Octavo of 224 pages, 35 illustrations. Philadelphia and London: W. B. Saunders Company, 1918. Published Bi-Monthly. Price, per year: Paper, $10.00; Cloth, $14.00.

This number is the sixth of the first volume, and is designated the Southern Number because the contributors are all representative practitioners of the Southern States. It consists of thirteen papers and while original research has been given a prominent place, the clinical subjects have by no means been neglected. Besides a general survey of the progress in pneumonias and tuberculosis, malaria is presented by an authority of large experience so that the knowledge imparted is more valuable than from the pen of a less experienced writer of the North. The careful paper on the study of ten pellagrins in two families living in the same neighborhood suggests the importance of considering the disease to be an infectious one.

A review of the index to the first volume of these clinics contains many reasons why these valuable publications should continue. The five previous numbers were named respectively the John's Hopkins number, the Philadelphia number, the New York number, the Boston number, and the Chicago number. All the articles upon timely subjects contributed are by some of our best known medical authors.

---

**The Hodgen Wire Cradle Extension Suspension Splint.** (The Exemplification of this Splint in the Treatment of Fractures and Wounds of the Extremities and Its Application in both Civil and War Practice). By Frank G. Nifong, M. D., F. A. C. S. With an Introduction by Harvey G. Mudd, M. D., F. A. C. S. Cloth, 162 pages, 124 illustrations. Price, $3.00. C. V. Mosby Company, St. Louis, 1918.

This small book opens with an introduction by Dr. Mudd, who feelingly writes of the life and works of John Thompson Hodgen, a man of unusual professional ability. He states that during the Civil War Hodgen's ingenuity combined the valuable features of the Nathan Ryno Smith and the Gurdon Buck suspense splints and produced his suspension splint that bears his name. While this brochure is supposedly limited to a discussion of The Hodgen Wire Cradle Suspension Splint it really devotes quite 100 pages to methods of splinting.

The author describes in detail many of the newer as well as the older forms of suspension and extension treatment of fractures. The book is freely illustrated adding greatly to the value of the text in that they accurately show important details. Treatment is given great consideration and none of the minutia that leads to a successful result are thought by the author too unimportant to describe. Furthermore, great stress is justly and wisely laid upon traction and extension as very important measures in certain types of injury. The monograph should be on the bookshelves of every up-to-date surgeon.

**Naval Hygiene.** By James Chambers Pryor, A. M., M. D. Medical Inspector, United States Navy; Master of Arts in Hygiene, Johns Hopkins University; Head of Department of Hygiene, U. S. Naval Medical School; Professor of Preventive Medicine, George Washington University. Cloth. Price, $3.00 net. Pp. 507, with 153 illustrations. Philadelphia: P. Blakiston's Son & Co.

Naval hygiene should be of special interest to the many doctors who today have enrolled for duty "on the trackless seas" as their "bit" of patriotism and loyalty, most of whom are totally ignorant of naval training and discipline. The author writes from the experience of years of naval service in all parts of the world and he hopes to make the way of the sea easier for others to comprehend. The comments on volunteers leaving ordinary civil land occupations and plunging into totally different surroundings are quite to the point. The author has set before us in a pleasing manner those essentials of Naval Hygiene which he considers will be of most value to physicians. He has stripped all irrelevant matter from the text and has written a compact, easily handled book. The greater number of illustrations almost without exception are splendid, well arranged and aptly illustrative of the point. The book contains much information that would enlighten even lay readers about the care of the health and wellbeing of the "tars" of "Uncle Sam." It would interest the thousands who have sons in our Navy. The book is well printed and free from typographical errors and is an excellent production of both printer and binder.

---

**Gynecology.** By William P. Graves, M. D., Professor of Gynecology at Harvard Medical School. Second Edition, Thoroughly Revised. Octavo volume of 883 pages with 490 original illustrations, 100 of them in colors. Philadelphia and London: W. B. Saunders Company, 1918. Cloth, $7.75 net.

To those of the medical profession familiar with the first edition the second edition of this valuable contribution to surgical literature needs no introduction. The work is designed both as a text-book and as a general reference book on the subpect of Gynecology. The estimable classification into three distinct parts is still retained. The subject matter has been brought down to date and includes a section on the Freudian theories of sex relation and its bearing on gynecological conditions. There have been many modifications in the text and several important revisions and additions have been made dealing with the relationship of gynecology to the internal secretions, the radium treatment of cancer, the radium therapy in non-malignant gynecologic diseases. In that part of the book devoted to operative gynecology a number of new operations have been described and illustrated. In fact, the illustrations which have been improved and which have been increased in number should be specially mentioned for they are extremely well executed by the well known artists Margaret Concree and Ruth Huetic. The gynecologist by studying them will be able critically to review his methods and results; while to the practitioner and student an opportunity is offered to consider the origin of the pathologic processes, the indications for operation and the well-advised management of gynecological diseases.

The volume is written in a style so simple and so clear that aside from its worth as a text-book, its perusal is distinctly a pleasure; it will be found of equal value to the practicing physician and to the expert gynecologist; its ownership is highly recommended to all practitioners whose practice requires a familiarity with conditions peculiar to woman.

---

**Nursing in Diseases of Children.** By Carl G. Leo-Wolf, M. D , Chief of Clinic for Sick Babies and Children for the Health Department of the City of Buffalo, N. Y.; Instructor in Pediatrics, University of Buffalo, Medical Department; Author of ''The Child in Health and Illness.'' Cloth, 314 pages, with 72 illustrations. Price, $2.50. St. Louis, C. V. Mosby Co., 1918.

The reviewer welcomes a book by a Buffalo author. Although it is written as a text-book for trained nurses it could fill an important place in disseminating knowledge in Nursing Children in Disease among mothers and even among physicians. The nurse who studies and retains the information found between the covers of this readable book would not only be better informed, but would be quite sure to be a greater help to the physician who often neglects the many important minor details in the conduct of the child's welfare.

The book opens in a short introductory chapter with a clear, simple exposition of the metric system of weights and measures which causes the nurse so much difficulty. Therein the author treats another perplexing problem of the nurse, that is the elements composing our foods and their caloric values.

The second chapter treats of the physiology of the child and discusses general considerations of the care of the child in such a manner as to simplify for the nurse the study of the development of the babe, infant and child. The section on feeding of children, disturbances of nutrition, and, diseases caused by malnutrition may well be commended not alone to the nurse but as well to the physician.

The chapter on clothing of the baby is a contribution by Miss M. J. Robertson, R. N., who has handled this trying subject with brevity and care, the tenor being toward simplicity in dress. The chapter on Public Health Nursing of Children, by Mrs. A. L. Hansen, R. N., who has treated the subject well, is most too brief.

The chapter on Mental Hygiene of Children is from the pen of H. G. Matzinger, M. D. This extremely important subject deserves special consideration by the reader. It alone would repay every parent to read this chapter as a guide to development and to character building of the child.

Instead of a careful compilation, the book, in the main, presents the most recent accredited information upon the various topics considered which treatment is quite apart from the personal touch from the ample experience of the author.

The author's style is conversational and familiar rather than the usual stilted and severely dignified one of the textbook, which in all makes this little work very readable.

---

**Surgical Treatment.** A Practical Treatise on the Therapy of Surgical Diseases for the use of Practitioners and Students of Surgery. By James Peter Warbasse, M. D., Formerly Attending Surgeon to the Methodist Episcopal Hospital, Brooklyn, New York. In three large octavo volumes, and separate Desk Index Volume. Volume I contains 947 pages with 699 illustrations. Philadelphia and London: W. B. Saunders Company, 1918. Per set (Three Volumes and the Index Volume): Cloth, $30.00 per set.

The author states in his preface that the three volumes of the work of which this is the first have been prepared primarily to "place in the hands of the surgeon the means for rendering help in every surgical condition under all circumstances" with the interest of the surgical patient always first in mind and to whom the work is dedicated. Every department is presented; not only general surgery, but special surgery. The brain, eye, nose, throat, skin, gynecology, genito-urinary, cosmetic, emergency surgery, minor surgery and bandaging. For the benefit of the medical student and general practitioner, he has purposely omitted all theoretical considerations presenting only essential and accepted facts in diagnosis and treatment.

The author states that as the work is written wholly by a single individual many other books have been consulted in its preparation, however he has not blindly followed the statements of other authors as is so often done in medical literature; whenever a method is described or considered that is still debatable the arguments are given but they are followed by the author's personal views.

The first volume is very broad in its scope; a discussion of the general principles of surgical treatment and surgical materials are first given. These chapters are along original lines in text-book composition and they will be of inestimable value, as they embrace the latest views of asepsis and antisepsis, anesthesia and anesthetics.

The chapter on "wounds and operations" discusses the various forms and their treatment sufficiently comprehensively. The chapter on "surgical fevers and infections" is thorough and very readable. The excellence of these chapters is but an index to the other chapters of this volume and demonstrate it to be one of the most valuable contributions to surgical literature.

The 2400 illustrations in the work are reproductions of photographs and clear drawings are used where photographs would be confusing. The illustrations add a great deal to the value of the work; they have been especially selected by the author and are well reproduced by the press work.

The thought in one's mind as he inspects the work is that in the preparation of it there must be a lack of originality but this is soon dispelled. The work has entailed an enormous amount of labor upon the author and too much credit cannot be given for the indefatigable energy with which the author has perused his task in the first volume in presenting the diversified material in so orderly a way.

---

**Principles and Practice of Infant Feeding.** By Julius H. Hess, M. D., Major M. R. C., U. S. Army, Active Service. Professor and Head of the Department of Pediatrics, University of Illinois College of Medicine; Chief of Pediatric Staff, Cook County Hospital; Attending Pediatrician to Cook County, Michael Reese and Englewood Hospitals, Chicago. Cloth, 338 pages, illustrated. Philadelphia: F. A. Davis Company, Publishers. English Depot, Stanley Phillips, London, 1918.

In this volume the author has given us a thoroughly scientific and up-to-date little book on the feeding of infants. Beginning with a chapter on metabolism he variously takes us through the care of the nursing infant, the premature infant, and finally the simple, rational feeding of milk mixtures according to tolerance. He then outlines the nutritional disturbances following the classification of Finkelstein and Czerny; this classification is the one now generally accepted and its appearance in this book marks the first in the English language to be well presented. Last, but not least, he points out the important relationship between infection and nutrition. It is an admirable book for both student and practitioner.

---

**The Orthopedic Treatment of Gunshot Injuries,** by Leo Mayer, M. D., Instructor in Orthopedic Surgery, New York Postgraduate Medical School and Hospital, with an introduction by Col. E. G. Brackett, M. C. N. A. Director of Military Orthopedic Surgery. 12mo of 250 pages, with 184 illustrations. Philadelphia and London: W. B. Saunders Company, 1918. Cloth, $2.50 net.

This little monograph brought out by the present great war has an introduction from the pen of Col. E. G. Brackett, M. C. N. A., who sets forth the marked value and usefulness of a work of this kind.

The author states that the book is not a treatise on orthopedic surgery, its purpose being "merely to emphasize certain principles and rules of guidance in the treatment of war injuries." Three years of military experience have given the author the opportunity to speak with some authority.

The book has the merit of great brevity without any loss of clarity or usefulness. With the aid of photographs and drawings the author emphasizes the importance of methods of treatment which will aid the surgeon as well as the specialist. This brief, very readable and instructive work should be carefully studied by all those whose medical duties will send them to cantonments or to the hospitals and stations back of the fighting lines.

